# Supplementary material for: Chromothripsis during telomere crisis is independent of NHEJ, and consistent with a replicative origin
Source: Genome Res. 2019 May;29(5):737–49. doi: 10.1101/gr.240705.118 (PMC6499312; doi:10.1101/gr.240705.118)
Supplement: Supplemental Material [file supp_gr.240705.118_Supplemental_file_1.zip › contigs/annotated_contigs/DB113/contig.3.DB113_length_281_mean_cov_6.48398576512.docx]

**DB113_length_281_mean_cov_6.48398576512**

```
AAATACAAAAATTAGTTGGGCATGGTGGCAGGCACCTGTAATCCAAGCTACTCTGGAGGCTGAGGTGGGAGAATCTTGAAACCAGGAGG
 >chr9:76547363-76547522 - E=2e-84
TGGAGGTTGCAGTGAGCAGAGATTGTGCCACTGCACTTCAGCCTGGCAACAGAGTGAGACTCCATCTCAA|CAA|CAACAACAACAACA
                                                                          >chr5:1044584
ACAACAACGACAACAACAAAAAAGCAGT|GAAGGAAAC|TGCCAGAATCATAGAGCTAAAAGTTGGTAGAATCAGGAATGAAATCTAGA
21-104458463 - E=6e-08                >chr10:31127354-31127423 + E=2e-13
ACCATAAGACAGCAAAGC
```
